# Intraokuläre Entzündung mit okklusiver retinaler Vaskulitis nach intravitrealer Brolucizumab-Injektion

**DOI:** 10.1007/s00347-021-01341-4

**Published:** 2021-02-24

**Authors:** Martin Dominik Leclaire, Jost Lauermann, Florian Alten, Nicole Eter

**Affiliations:** Klinik für Augenheilkunde, Universitätsklinikum Münster, Domagkstr. 15, 48149 Münster, Deutschland

## Anamnese und klinischer Befund

Ein 77-jähriger Patient mit bekannter neovaskulärer altersabhängiger Makuladegeneration ([nAMD], Erstdiagnose und Initiierung einer Anti-vascular-endothelial-growth-factor[VEGF]-Therapie im Dezember 2013) stellte sich 3 Tage nach seiner zweiten intravitrealen Injektion mit Brolucizumab (IVB) (73 Tage nach der ersten IVB) notfallmäßig mit Schmerzen und Visusverschlechterung am rechten Auge in unserer Ambulanz vor.

**Figure 1:**
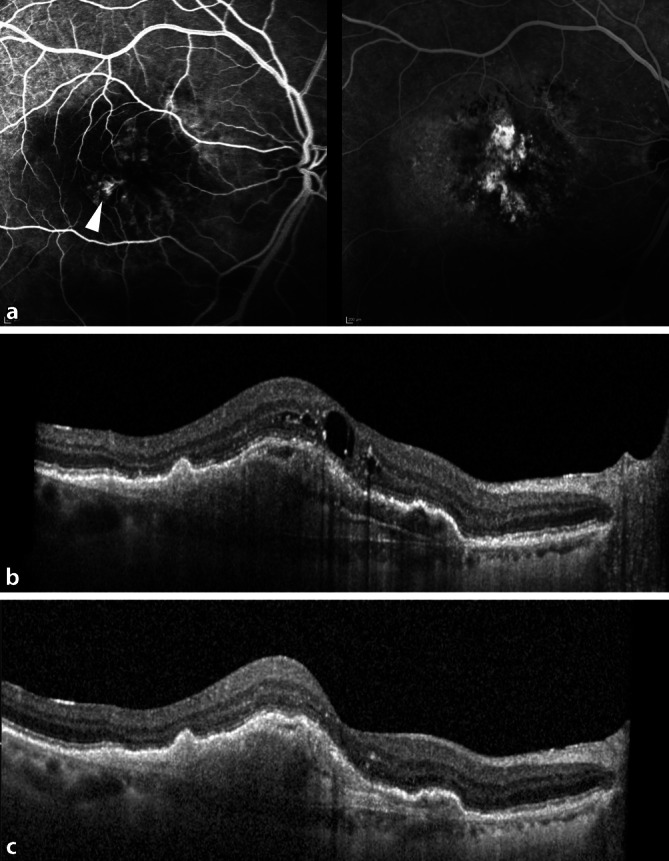

Der Patient hatte in der Vergangenheit multiple intravitreale Injektionen am rechten Auge erhalten. Aufgrund der hohen, fast monatlichen Injektionsfrequenz war bei dem Patienten im April 2020 bei erneuten Aktivitätszeichen bei nAMD erstmalig eine IVB erfolgt (Abb. 1a, b), die eine gute Wirksamkeit zeigte (Abb. 1c). Bei der zuletzt durchgeführten Untersuchung 4 Wochen nach erster IVB zeigten sich eine komplette Regression der intra- und subneurosensorischen Flüssigkeit und ein Visus von 0,4. Hinweise auf eine intraokulare Entzündung oder eine okklusive retinale Vaskulitis ergaben sich zu diesem Zeitpunkt nicht, der Patient beklagte keinerlei Beschwerden.

**Figure 2:**
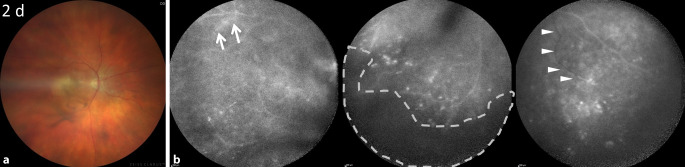

Bei der notfallmäßigen Vorstellung betrug der bestkorrigierte Visus rechts 1/15 Metervisus, der intraokulare Druck war normoton. Spaltlampenbiomikroskopisch zeigten sich rechts Endothelbeschläge im Arlt-Dreieck und Zellen in der Vorderkammer. Weder eine Bindehautinjektion noch ein Hypopyon waren sichtbar. Fundoskopisch ergab sich ein reduzierter Einblick bei Glaskörperschlieren und -zellen; soweit einsehbar stellten sich eine randscharfe, vitale Papille, makulär eine vorbekannte Pigmentepithelabhebung und Pigmentepithelverschiebungen (Abb. 2a) sowie peripher der Verdacht auf retinale Okklusionen dar. Die 55°-Fluoreszeinangiographie (FLA) ergab perivaskuläre Leckagen, periphere Gefäßabbrüche und diffuse hyperfluoreszente Spots (Abb. 2b).

## Diagnose

Wir stellten die Diagnose einer intraokularen Entzündung mit okklusiver retinaler Vaskulitis nach IVB.

## Therapie und Verlauf

Der Patient wurde stationär aufgenommen und eine systemische intravenöse (i.v.) Therapie mit Kortikosteroiden gewichtsadaptiert begonnen (Prednisolon 80 mg/Tag).

**Figure 3:**
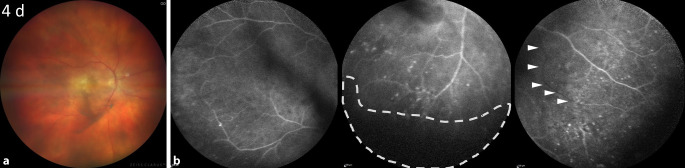

Im weiteren Verlauf nahmen der Vorderkammer- und Glaskörperreiz langsam ab, und der Visus erholte sich innerhalb von 4 Tagen bis auf bestkorrigiert 0,125 bei gebessertem Funduseinblick (Abb. 3a). Bei der erneut durchgeführten 55°-FLA zeigten sich peripher eine abnehmende phlebitische Leckage und persistierende okklusive Gefäße (Abb. 3b).

**Figure 4:**
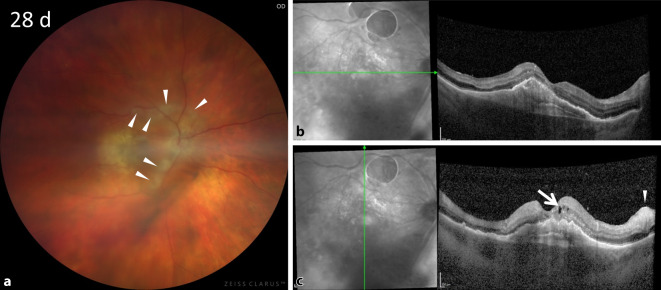

Es erfolgten engmaschige ambulante Verlaufskontrollen, die ein weiteres Abnehmen des Vorderkammer- und Glaskörperreizes und ein Aufklaren des Funduseinblickes ergaben. Die orale Kortikosteroiddosis wurde stufenweise reduziert. Bei einer Verlaufskontrolle 28 Tage nach IVB imponierten fundoskopisch demarkierte Areale im Bereich der großen Gefäßbögen (Abb. 4a). Korrespondierend stellte sich kohärenztomographisch eine am ehesten ischämisch bedingte Schwellung der inneren Netzhautbanden mit Aussparung der Fovea dar (Abb. 4b).

## Diskussion

Brolucizumab (Beovu, Novartis, Basel, Schweiz) ist seit Februar 2020 in der Europäischen Union zur Behandlung der exsudativen AMD zugelassen [9]. Der Vorteil im Vergleich zu bereits verfügbaren Anti-VEGF-Wirkstoffen liegt in einer möglichen verlängerten Wirkdauer und damit in einer Verringerung der Injektionsfrequenz, die in den Zulassungsstudien HAWK und HARRIER demonstriert werden konnten [4].

In der klinischen Anwendungspraxis konnten in ersten Real-world-Studien eine gute Wirksamkeit und eine mögliche Ausweitung der Injektionsintervalle bestätigt werden [1, 7]. Auch in dem hier beschriebenen Fall führte die erstmalige IVB zu einer vollständigen Flüssigkeitsresorption (Abb. 1b, c), was mit den vorhergegangenen Injektionen nicht erreicht werden konnte.

Mittlerweile liegen Fallberichte vor, die eine intraokulare Entzündung mit okklusiver retinaler Vaskulitis nach IVB thematisieren. Novartis rief als Reaktion auf die berichteten Nebenwirkungen ein Komitee (*Safety Review Committee*) zur Aufarbeitung und Bewertung der Fälle ins Leben [6] Darüber hinaus wurde im September 2020 die Fachinformation im Hinblick auf das mögliche Auftreten einer okklusiven retinalen Vaskulitis ergänzt [10].

Kürzlich wurden Handlungs- und Therapieempfehlungen hinsichtlich des Auftretens einer intraokularen Entzündung mit okklusiver Vaskulitis nach IVB publiziert [2]. Hier wird die dezidierte präoperative Aufklärung der Patienten über mögliche Symptome empfohlen wie die Wahrnehmung von Floatern, okuläre Missempfindungen oder zusätzlich verschwommenes und verschlechtertes Sehen mit ggf. Gesichtsfeldausfällen. Auch wird die Wichtigkeit der Differenzierung zwischen einer intraokularen Entzündung und einer exogenen Endophthalmitis betont.

Im hier vorgestellten Fall äußerte der Patient nur unspezifische Symptome. Daher waren der klinische Befund mit fehlendem Hypopyon und die Fluoreszeinangiographie mit den dargestellten Gefäßabbrüchen richtungsweisend.

Als weitere Handlungsempfehlung wird in der Expertenmeinung [2] die Anwendung von lokalen oder systemischen Kortikosteroiden und die Beendigung der Brolucizumab-Therapie ausgesprochen. Ferner wird die Bedeutung der multimodalen Bildgebung zur Diagnostik hervorgehoben.

Baumal et al. beschreiben in einer Fallserie eine mittlere Dauer von 30 Tagen nach IVB bis zur Diagnose einer retinalen Vaskulitis [3]. In unserem Fall ereignete sich die Vaskulitis untypischerweise bereits 3 Tage nach der zweiten IVB und 73 Tage nach der ersten IVB.

Witkin et al. verweisen im Hinblick auf die Ätiologie der Vaskulitis auf die im Rahmen der Zulassungsstudien erhobenen Daten zum Vorliegen von Anti-Brolucizumab-Antikörpern und leiten einen Erklärungsansatz für das Auftreten der okklusiven retinalen Vaskulitiden auch aus einem möglichen vasokonstriktiven Effekt ab [8]. Aufgrund des zeitlichen Abstandes des Beschwerdebeginns zur IVB wird in Fallberichten als Ursache für die intraokuläre Entzündung mit retinaler Vaskulitis eine verzögerte Hypersensitivitätsreaktion (Typ-IV-Hypersensitivitätsreaktion) auf den Wirkstoff diskutiert [5].

In dem hier vorgestellten Fall waren 4 Wochen nach zweiter IVB perivaskuläre Ischämien an den großen Gefäßbögen sichtbar (Abb. 4a–c), die darauf hindeuten, dass die Okklusionen nicht nur die peripheren Arteriolen, sondern im Verlauf auch zentrale Gefäße betroffen haben.

## Zusammenfassung

Brolucizumab erscheint auf Grundlage der Zulassungsstudien und der ersten Real-world-Erfahrungen als ein vielversprechender Anti-VEGF-Wirkstoff für AMD-Patienten mit hoher Injektionslast. Brolucizumab scheint mit einem höheren Risiko für eine intraokulare Entzündung mit okklusiver retinaler Vaskulitis verbunden zu sein als die bereits länger verfügbaren Anti-VEGF-Präparate. Eine solche kann auch nach vorausgegangenen komplikationsfreien Brolucizumab-Injektionen auftreten und retinale Gefäße verschiedenen Kalibers betreffen. Der Zeitpunkt des Auftretens nach IVOM, der klinische Befund und insbesondere die FLA mit Erfassung der Netzhautperipherie sind wegweisend bei der Diagnosestellung und Abgrenzung der okklusiven Vaskulitis von der exogenen Endophthalmitis nach Brolucizumab-Injektion. Patienten müssen auf das Risiko einer intraokularen Entzündung mit retinaler okklusiver Vaskulitis vor Brolucizumab-Injektion hingewiesen werden.
